# Multiple metabolic analysis of [^18^F]FDG PET/CT in patients with kidney disease

**DOI:** 10.1016/j.heliyon.2025.e42522

**Published:** 2025-02-06

**Authors:** Hao Jiao, Yongkang Qiu, Zhao Chen, Yongbai Zhang, Wenpeng Huang, Qi Yang, Lei Kang

**Affiliations:** Department of Nuclear Medicine, Peking University First Hospital, Beijing, 100034, China

**Keywords:** Renal function, Kidney disease, [^18^F]FDG, PET/CT, Metabolic parameter

## Abstract

**Purpose:**

This study aimed to evaluate the value of [^18^F]FDG PET/CT in patients with kidney disease by using multiple metabolic parameters.

**Materials and methods:**

A retrospective review of 182 kidney disease patients and 32 controls was conducted. Patients were categorized into acute kidney disease (AKD), AKI on CKD (A/C), and chronic kidney disease (CKD) groups, further divided by CKD stage and disease etiology. Regions of interest (ROIs) were drawn in renal cortex, liver, aorta, and lesions. SUVmax and SUVmean were measured, and ratios of renal cortex SUVmax to liver and blood pool SUVmean were calculated.

**Results:**

Abnormal FDG uptake was observed in 84.6 % of patients, with significantly higher SUVmax in malignant versus benign lesions. Common malignancies included multiple myeloma, lymphoma, and lung cancer. PET/CT had 89.5 % sensitivity and 100 % specificity for tumor detection. SUVs differed significantly among AKD, A/C, CKD, and normal groups. Significant differences in SUVmax and SUVmean were also found between CKD stages and primary versus secondary kidney diseases. In CKD, increased SUVmax and SUVmean correlated with lower serum creatinine and blood urea nitrogen, and increased eGFR.

**Conclusion:**

For patients with kidney disease, [^18^F]FDG PET/CT can be used to systematically screen tumors and inflammatory lesions. And the [^18^F]FDG uptake of renal cortex may distinguish different types of kidney diseases and is correlated with renal function.

## 1 Introduction

Kidney disease is defined as abnormalities in kidney structure or function, with implications for health. Kidney Disease: Improving Global Outcomes (KDIGO) proposed the term acute kidney disease (AKD) to define any acute condition that impacts kidney function including acute kidney injury (AKI), estimated glomerular filtration rate (eGFR) < 60 mL/min/1.73 m^2,^ a decrease in GFR by > 35 %, an increase in serum creatinine of >50 %, or any kidney damage lasting <3 months. Chronic kidney disease (CKD) is defined by the persistence of kidney disease for a period of >90 days [1]. For patients with pre-existing CKD, the AKI event can be superimposed on CKD, and this kind of patients is defined as AKI superimposed on CKD (A/C) [2].

Kidney disease appears to be both a potential cause and consequence of tumor. Kidney dysfunction creates an inflammatory microenvironment and oxidative stress, thus providing an ideal environment for the development of tumor [3]. It has been proved that patients with end-stage renal disease have a higher risk of developing tumor than individuals with normal renal function [4]. Patients with tumor often have kidney problems. In addition to direct tumor infiltration of the kidney, the causes of kidney disease in patients with tumor include the use of chemotherapeutic drugs, paraneoplastic glomerulopathies, tumor triggering malignant ureteral obstruction, etc [3,5]. Therefore, for patients with kidney disease, timely detection of kidney-associated tumors and exclusion of other local or systemic tumors are particularly important.

Positron emission tomography/computed tomography (PET/CT) combines the functional and metabolic information provided by PET with the anatomical information of CT, and it is widely used for lesion localization, auxiliary diagnosis and efficacy assessment [6,7]. [^18^F]fluorodeoxyglucose ([^18^F]FDG) is a structural analogue of 2-deoxyglucose, which is most commonly used in daily clinical practice and high FDG uptake can be observed in tissues with increased glucose metabolism, including malignant and inflammatory tissues, as [^18^F]FDG is transported into cells via glucose transporter proteins. In addition, [^18^F]FDG is excreted mainly through urine, leading to accumulation in the kidney and urinary tract [8]. However, it has not been demonstrated by any studies yet whether detecting [^18^F]FDG uptake in the renal cortex through PET/CT can provide relevant clinical information. In this study, we aimed to evaluate the value of [^18^F]FDG PET/CT in patients with kidney disease, including tumor and inflammation.

## 2 Material and methods

### 2.1 Patients

**Fig. 1:**
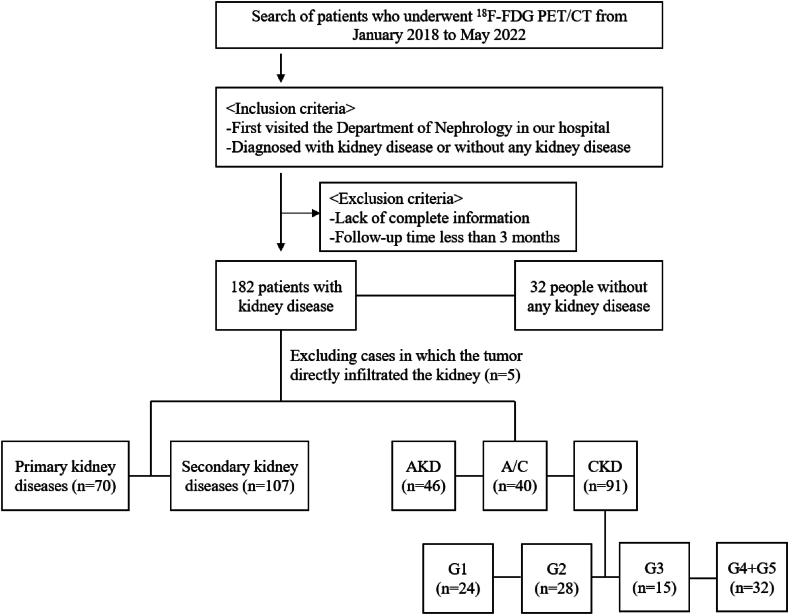
Flowchart of patient selection for this retrospective study. The chronic kidney disease groups were categorized based on estimated glomerular filtration rate (eGFR) values calculated using the CKD-EPI equation as follows: G1 (eGFR ≥90 mL/min/1.73 m^2^, n = 24), G2 (eGFR 60–89 mL/min/1.73 m^2^, n = 28), G3 (eGFR 30–59 mL/min/1.73 m^2^, n = 15), and G4+G5 (eGFR <30 mL/min/1.73 m^2^, with G4 defined as eGFR 15–29 mL/min/1.73 m^2^ and G5 as eGFR <15 mL/min/1.73 m^2^, n = 24). AKD, acute kidney disease; CKD, chronic kidney disease; A/C, acute kidney injury superimposed on chronic kidney disease.

We retrospectively reviewed 182 [^18^F]FDG PET/CT subjects diagnosed with kidney disease and 32 subjects without any kidney disease from January 2018 to May 2022 in Peking University First Hospital. These patients with kidney disease first visited the Department of Nephrology and underwent further [^18^F]FDG PET/CT examination because of increased tumor markers, or the discovery of lymph node enlargement, or fever of unknown origin, which required screening for tumor lesions or finding infectious lesions. All patients underwent detailed clinical examinations. The documented clinical indicators included age, gender, creatinine, urea nitrogen, eGFR, pathology findings and results of PET/CT. The pathology findings included pathological results of kidney and other sites (if any). They were also followed up from 3 months to 4 years (median duration of 2 years and 9 months). Patients were excluded if they lacked the above clinical information or the follow-up time was less than 3 months. As for the study of renal cortex uptake, we excluded 5 cases in which the tumor directly infiltrated the kidney. According to the course of the disease and the history of kidney disease, patients with kidney disease were divided into three groups: AKD (n = 46), A/C (n = 40), and CKD (n = 91). The CKD groups were categorized based on eGFR values calculated using the chronic kidney disease epidemiology collaboration (CKD-EPI) equation as follows: G1 (eGFR ≥90 mL/min/1.73 m^2^, n = 24), G2 (eGFR 60–89 mL/min/1.73 m^2^, n = 28), G3 (eGFR 30–59 mL/min/1.73 m^2^, n = 15), and G4+G5 (eGFR <30 mL/min/1.73 m^2^, with G4 defined as eGFR 15–29 mL/min/1.73 m^2^ and G5 as eGFR <15 mL/min/1.73 m^2^, n = 24) (Fig. 1).

### 2.2 [^18^F]FDG PET/CT imaging

The patient was fasted for at least 6 h and avoided strenuous exercise. The blood glucose level was measured before FDG administration to ensure that the patient's blood glucose was below 11 mmol/L [^18^F]FDG PET/CT (Philips GXL-16 PET/CT, Version 3.5) scan was performed 60–80 min after the administration of [^18^F]FDG 3.7 MBq/kg by intravenous, provided by Beijing Atom high-tech Co., Ltd. and radiochemical purity >95 %. Low-dose CT scanning was performed with a tube voltage of 120 kV, a tube current of 100 mA/s, using soft tissue reconstruction algorithm with a layer thickness of 2 mm. The PET scanning ranged from the cranial top to the middle of the thigh, including the limbs if necessary, and was collected for 1.5 min per bed. The images were reconstructedusing ordered subset expectation maximization.

**Fig. 2:**
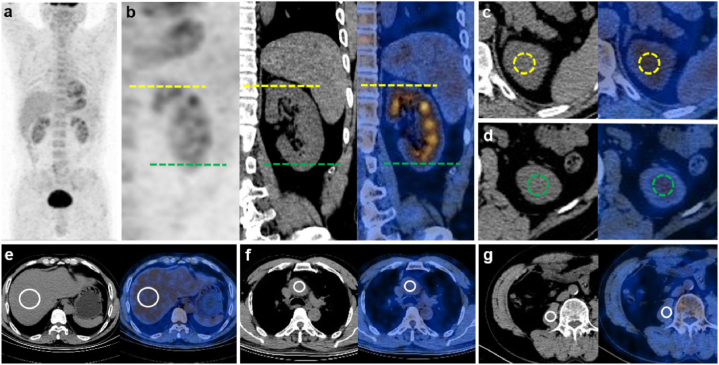
Example of the measurement of SUVmax for renal cortex, SUVmean for renal cortex, liver, blood pool and psoas muscle by [^18^F]FDG PET/CT. ROI drawing was performed accordingly (**a**) The whole-body maximum intensity projection. (**b**) Coronal images of the left kidney, yellow intermittent lines showing the position of upper pole of renal cortex, and green intermittent lines showing the position of lower pole of renal cortex. (**c–d**) Transverse images of the upper and lower pole of renal cortex, the ROI regions were shown as the yellow and green circles, respectively. Transverse images of liver (**e**), blood pool (**f**) and psoas muscle (**g**), the ROI regions were shown as the white circles.

The [^18^F]FDG PET/CT scans were reviewed by two blinded and independent nuclear medicine physicians. For semi-quantitative analysis, two regions of interest (ROIs) were drawn in the left renal cortex of both upper and lower poles (100 mm^2^). In patients with CT-confirmed lesions with higher [^18^F]FDG uptake than the background of the surrounding tissue, the ROI of the lesion was also drawn. Additional ROIs were drawn in central region of the right lobe of the liver (500 mm^2^), terminal part of ascending aorta (200 mm^2^) and right psoas muscle at the third lumbar skeletal level (100 mm^2^), which are generally considered as homogeneous baseline [^18^F]FDG radioactive regions. The ROI for the left kidney was evaluated visually in both transverse and coronal planes to ensure the ROI was located in the cortex and away from the urine. The ROI for other organs was placed on transverse PET/CT images (Fig. 2a–g). According to the PET/CT images, the SUVmax and SUVmean were measured for each ROIs, in which the [^18^F]FDG uptake value of renal cortex was the average of the [^18^F]FDG uptake values of upper and lower poles. The ratio of renal cortex SUVmax to the liver SUVmean (kidney/liver uptake ratio), the ratio of renal cortex SUVmax to the blood pool SUVmean (kidney/blood uptake ratio) and the ratio of renal cortex SUVmax to the psoas muscle SUVmean (kidney/muscle uptake ratio) were obtained.

### 2.3 Statistical analyses

Data were expressed as mean ± standard deviation and as median (range). Mann-Whitney *U* test and Kruskal-Wallis test with Bonferroni correction were used to compare the [^18^F]FDG uptake among groups. The correlation between laboratory analysis and renal cortex uptake was assessed using the Spearman relation analysis. If there was a significant difference in [^18^F]FDG uptake between two groups, the receiver operating characteristic (ROC) analysis was performed to determine the AUC value. Sensitivity and speciﬁcity were extrapolated from the ROC curve, targeting sensitivity as close to 1 as probable. All statistical analyses were performed using the SPSS Statistics software program (version 26.0; IBM, Chicago, IL, USA) and the GraphPad Prism 9 software. *P* < 0.05 was considered to represent a statistically significant difference.

## 3 Results

### 3.1 Clinical features

This study reviewed 182 patients with kidney disease and 32 controls without any kidney disease. Details of the patients were shown in Table 1. There was no significant difference in age (*P* = 0.797) and gender (*P* = 0.403) between the two groups. There were 111 males (61 %) and 71 females (39 %) among the patients with kidney disease, with a median age of 59 years (range 17–86 years). The median serum creatinine and urea nitrogen in the patients with kidney disease were 150.5 μmol/L (range 41–1867 μmol/L) and 10.54 mmol/L (range 2.58–44.29 mmol/L), compared to 73 μmol/L (range 48.4–90.8 μmol/L) and 4.92 mmol/L (range 2.29–7.05 mmol/L) in the control group, respectively. The serum creatinine (*P* < 0.0001) and urea nitrogen (*P* < 0.0001) showed significant differences between the two groups.

**Table 1:** Clinical characteristics of patients with or without kidney disease.

Characteristic	With kidney disease	Without kidney disease	*P*
Number	182	32	
Age (year)			0.797
Median	59	59	
Range	17–86	29–87	
Gender [No. (%)]			0.403
Male	111 (61 %)	17 (53 %)	
Female	71 (39 %)	15 (47 %)	
Serum creatinine (μmol/L)			<0.0001
Median	150.5	73	
Range	41–1867	48.4–90.8	
Blood urea nitrogen (mmol/L)			<0.0001
Median	10.54	4.92	
Range	2.58–44.29	2.29–7.05	

According to the clarity of disease etiology, all kidney disease patients could be classified into primary kidney disease (n = 70) and secondary kidney disease (n = 112), with the causes of secondary kidney diseases listed in Table 2. The three most common causes were autoimmune and connective tissue diseases (n = 32), paraproteinemia (n = 31) and tumor (n = 25). “Others” in the table included hypertensive nephropathy, obesity related glomerulopathy, etc. There were 11 patients in this category. Additional causes of secondary kidney disease were metabolic disorder (n = 8), IgG4-related diseases (n = 3), infection (n = 1) and drugs (n = 1).

**Table 2:** The etiology of secondary kidney disease.

Etiology	Number of patients
Autoimmune and connective tissue diseases	32
Tumor	25
Paraproteinemia	31
Infection	1
Metabolic disorder	8
IgG4-related diseases	3
Drugs	1
Others	11

### 3.2 Abnormal [^18^F]FDG uptake detected by PET/CT in patients with kidney disease

Abnormal increase of [^18^F]FDG uptake was found in 154 of 182 patients (84.6 %), of which 33 cases were malignant lesions, 118 cases were benign lesions and 3 cases could not be identified. The three most common malignant lesions included multiple myeloma, lymphoma and lung cancer, while the three most common benign lesions included inflammatory lesions, physiological uptake and autoimmune lesions. Malignant lesions had a median SUVmax of 6.3 (range 2.5–16), whereas benign lesions had a median SUVmax of 4.25 (range 1.2–13.3), and there was a significant difference between the two groups (*P* < 0.001). In addition to the malignancies with increased [^18^F]FDG uptake, there was one case myeloma with normal uptake. A total of 34 malignancies were diagnosed by PET/CT, with 4 cases missed, resulting in an overall sensitivity of 89.5 %. Of these 4 missed cases, two were multiple myeloma and two were chronic lymphocytic leukemia.

### 3.3 Comparison of tumors related and unrelated to kidney disease

Tumor that we considered related to kidney disease included multiple myeloma, tumors that cause lower urinary tract obstruction, and tumors that directly invade the kidney. So malignant tumors could be divided into two types, one related to kidney disease and the other unrelated to kidney disease. The former had a median SUVmax of 6.5 (range 2.5–16), while the latter had a median SUVmax of 5.85 (range 2.9–14.8), without significance between the two groups (*P* = 0.75). Tumors related to kidney disease included multiple myeloma (n = 17), lymphoma (n = 6), cervical cancer (n = 1) and renal pelvis and ureter cancer (n = 1). Tumor unrelated to kidney disease included lung cancer (n = 3), lymphoma (n = 2), thyroid cancer (n = 1), liver cancer (n = 1), etc. Details were shown in Table 3.

**Table 3:** Characteristics of tumors detected by [^18^F]FDG PET/CT.

Relation between kidney disease and tumor	Tumor type	Number of cases	Number of tumors detected by PET/CT	Mean SUV value of tumors with increased uptake (range)
Tumor-related to kidney disease	Lymphoma	6	5	10.6 ± 5.0 (2.90–16.0)
	Multiple myeloma	17	15 (including a case of normal uptake)	6.1 ± 3.2 (2.5–12.5)
	Cervical cancer	1	1	7.9
	Renal pelvis and ureter cancer	1	1	7.9
Tumor-unrelated to kidney disease	Thyroid cancer	1	1	4.6
	Thymic cancer	1	1	2.9
	Lung cancer	3	3	4.7 ± 1.5 (3.3–6.3)
	Liver cancer	1	1	6.5
	Gastric cancer	1	1	5.4
	Colon cancer	1	1	14.6
	Lymphoma	2	1	6.5
	Ovarian cancer	1	1	6.9
	Fallopian tube cancer	1	1	14.8
	Breast cancer	1	1	5

### 3.4 Comparison of patients in AKD, A/C, CKD and normal groups

**Fig. 3:**
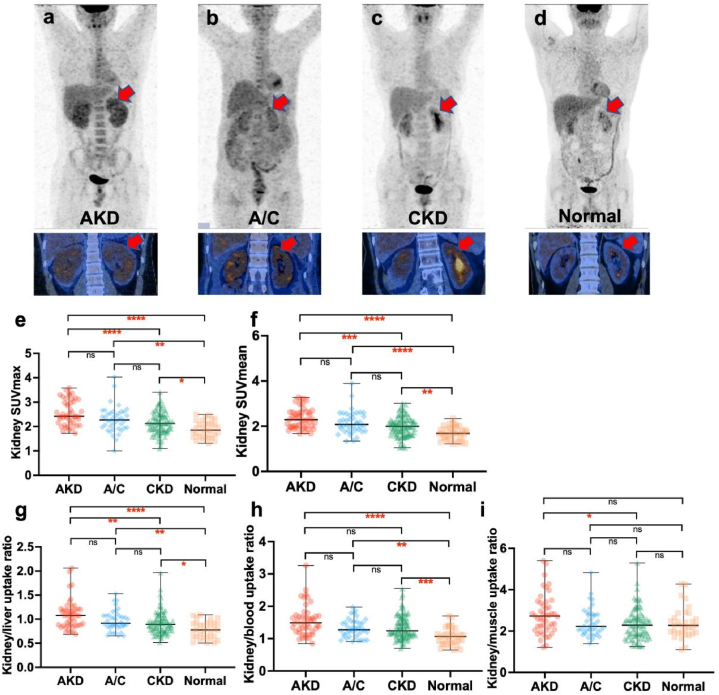
Comparison of renal cortex uptake among acute kidney disease (AKD), acute kidney injury superimposed on chronic kidney disease (A/C), chronic kidney disease (CKD) and normal groups (**a–d**) Coronal images of renal cortex in the four groups (red arrow). (**e–i**) Statistical images of comparison of renal cortex SUVmax, SUVmean, kidney/liver uptake ratio, kidney/blood uptake ratio and kidney/muscle uptake ratio among the four groups.

The renal cortex SUVmax, SUVmean, kidney/liver uptake ratio and kidney/blood uptake ratio in AKD, A/C and CKD groups were significantly higher than those in the normal group. And there were significant differences in renal cortex SUVmax, SUVmean and kidney/liver uptake ratio between AKD and CKD groups. For kidney/muscle SUVmax, however, only AKD and CKD groups had significant differences (*P* = 0.041) (Fig. 3a–i). Detailed SUV values are summarized in Table 4.

**Table 4:** Median SUV value (minimum-maximum) of renal cortex in different kidney diseases groups.

	AKD (n = 46)	A/C (n = 40)	CKD (n = 91)	Normal (n = 32)	CKD G1 (n = 24)	CKD G2 (n = 28)	CKD G3 (n = 15)	CKD G4+G5 (n = 32)	Primary kidney disease (n = 70)	Secondary kidney disease (n = 107)
Kidney SUVmax	2.42 (1.72–3.58)	2.27 (1.00–4.03)	2.12 (1.10–3.41)	1.85 (1.31–2.50)	2.37 (1.41–3.06)	2.14 (1.35–2.96)	2.03 (1.23–2.49)	1.90 (1.10–3.41)	2.13 (1.00–3.06)	2.35 (1.10–4.03)
Kidney SUVmean	2.30 (1.67–3.27)	2.08 (1.34–3.02)	1.99 (1.05–3.02)	1.68 (1.22–2.34)	2.19 (1.06–2.77)	2.01 (1.23–2.83)	1.86 (1.09–2.96)	1.78 (1.22–2.34)	2.01 (1.06–2.78)	2.17 (1.05–3.89)
Kidney/liver uptake ratio	1.08 (0.69–2.06)	0.92 (0.65–1.53)	0.89 (0.51–1.97)	0.78 (0.51–1.09)	0.96 (0.60–1.45)	0.86 (0.68–1.62)	0.77 (0.51–1.01)	0.89 (0.62–1.97)	0.87 (0.60–1.31)	1.02 (0.51–2.06)
Kidney/blood uptake ratio	1.49 (0.85–3.26)	1.27 (0.91–1.97)	1.24 (0.70–2.55)	1.07 (0.65–1.70)	1.38 (0.97–2.28)	1.19 (0.93–2.36)	1.16 (0.77–2.55)	1.30 (0.70–2.00)	1.23 (0.86–2.55)	1.40 (0.70–3.26)
Kidney/muscle uptake ratio	2.72 (1.22–5.41)	2.23 (1.39–4.83)	2.28 (1.25–5.30)	2.28 (1.10–4.27)	2.60 (1.28–4.04)	2.36 (1.25–3.57)	1.92 (1.29–3.17)	2.04 (1.26–5.30)	2.23 (1.22–3.61)	2.48 (1.26–5.41)
Liver SUVmean	2.42 (1.08–3.44)	2.39 (1.72–3.50)	2.23 (1.60–3.51)	2.38 (1.40–3.25)	2.26 (1.67–3.37)	2.42 (1.67–3.51)	2.54 (1.92–3.13)	1.97 (1.60–3.12)	2.47 (1.67–3.51)	2.35 (1.08–3.44)
Blood pool SUVmean	1.69 (0.66–3.09)	1.75 (1.24–2.69)	1.62 (0.83–2.86)	1.84 (1.11–2.52)	1.65 (1.18–2.86)	1.70 (1.06–2.47)	1.59 (0.83–2.01)	1.44 (0.91–2.69)	1.87 (0.83–2.86)	1.81 (0.85–3.18)
Muscle SUVmean	0.96 (0.58–1.48)	0.95 (0.51–1.82)	0.93 (0.54–1.51)	0.81 (0.51–1.42)	0.93 (0.61–1.51)	0.96 (0.61–1.40)	0.97 (0.69–1.44)	0.89 (0.54–1.35)	0.97 (0.61–1.44)	0.92 (0.51–1.82)

AKD, acute kidney disease; CKD, chronic kidney disease; A/C, acute kidney injury superimposed on chronic kidney disease.

### 3.5 Comparison of patients with different CKD stages

**Fig. 4:**
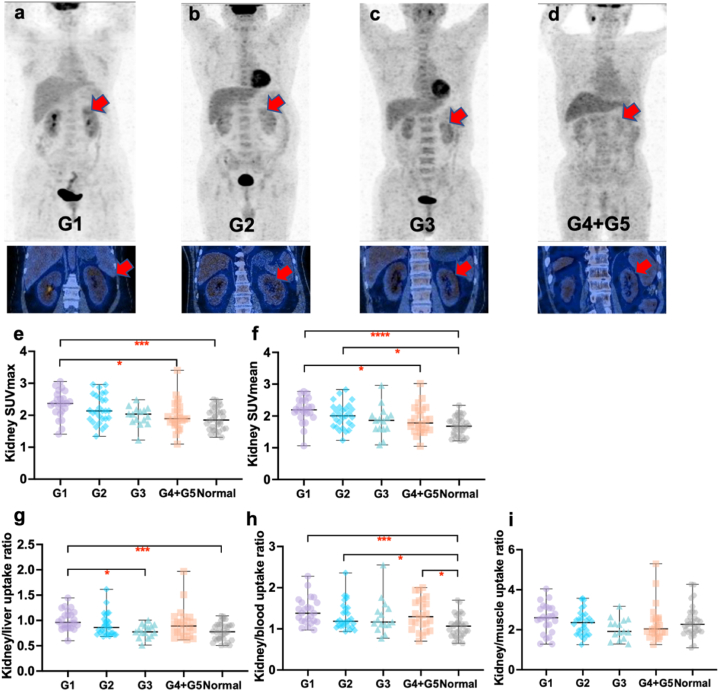
Comparison of renal cortex uptake among normal and different chronic kidney disease stages groups (**a–d**) Coronal images of renal cortex in different chronic kidney disease groups (red arrow). (**e–i**) Statistical images of comparison of renal cortex SUVmax, SUVmean, kidney/liver uptake ratio, kidney/blood uptake ratio and kidney/muscle uptake ratio among the five groups.

The SUVmax, SUVmean, kidney/liver uptake ratio and kidney/blood uptake ratio in G1 group were all significantly higher than those in normal group. Significant difference in kidney/blood SUVmax was also observed between G2 and normal groups, as well as between G4+G5 and normal groups. We also observed significant differences between G1 group and G3 or G4+G5 group in other indicators (Fig. 4a–i). Detailed SUV values are summarized in Table 4.

### 3.6 Comparison of patients with primary and secondary kidney disease

**Fig. 5:**
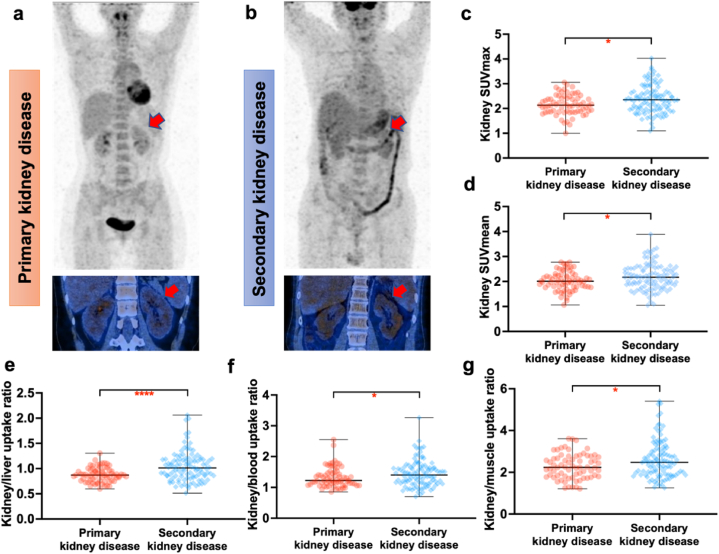
Comparison of renal cortex uptake between primary and secondary kidney disease groups (**a–b**) Coronal images of renal cortex in primary and secondary kidney disease group (red arrow). (**c–g**) Statistical images of comparison of renal cortex SUVmax, SUVmean, kidney/liver uptake ratio, kidney/blood uptake ratio and kidney/muscle uptake ratio between the two groups.

According to the clarity of disease etiology, patients with kidney disease were divided into primary kidney disease (n = 70) and secondary kidney disease (n = 107). The SUVmax, SUVmean, kidney/liver uptake ratio, kidney/blood uptake ratio and kidney/muscle uptake ratio of patients with secondary kidney disease were all significantly higher than those with primary kidney disease, with the most pronounced difference observed in kidney/liver uptake ratio (*P* < 0.0001) (Fig. 5a–g). Detailed SUV values are summarized in Table 4.

### 3.7 Comparison of [^18^F]FDG uptake in liver, blood pool and muscle among groups

**Fig. 6:**
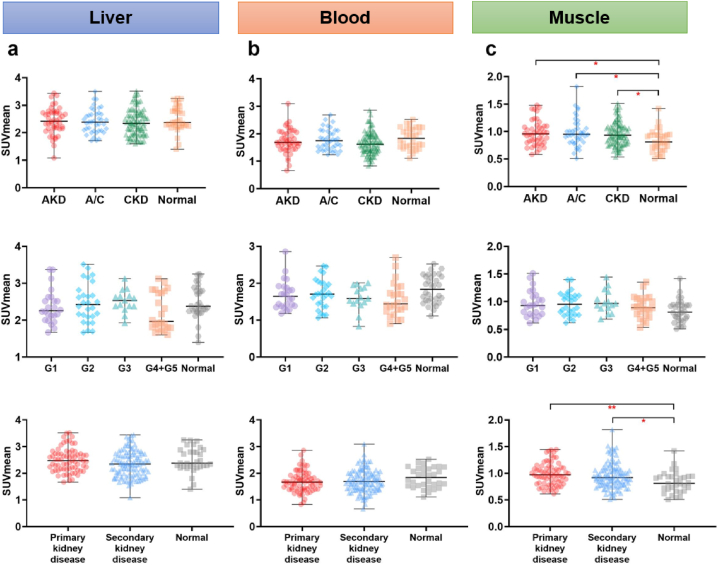
The comparison of SUVmean of liver (**a**), blood pool (**b**) and muscle (**c**) among different groups. AKD, acute kidney disease; CKD, chronic kidney disease; A/C, acute kidney injury superimposed on chronic kidney disease.

No significant difference was observed in liver (*P* = 0.14), blood pool (*P* = 0.09), or psoas muscle (*P* = 0.06) SUVmean among normal and different CKD stages groups. There was also no significant difference in liver (*P* = 0.55) or blood pool (*P* = 0.09) SUVmean observed between AKD, A/C or CKD groups and the normal group. Likewise, neither primary nor secondary group exhibited a significant difference in liver (*P* = 0.09) or blood pool (*P* = 0.18) SUVmean compared to the normal group. However, psoas muscle was significantly higher in AKD (*P* = 0.03), A/C (*P* = 0.04) and CKD (*P* = 0.04) compared to the normal group, as well as in primary kidney disease (*P* < 0.01) and secondary kidney disease (*P* = 0.03) (Fig. 6a–c). Detailed SUV values are summarized in Table 4.

### 3.8 Correlation between laboratory analysis and renal cortex uptake in CKD group

**Fig. 7:**
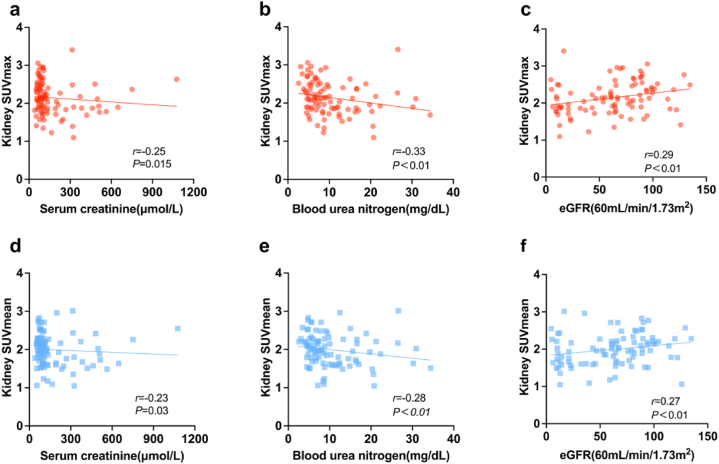
Correlation studies between renal cortex SUVmax and serum creatinine (**a**), urea nitrogen (**b**), and estimated glomerular filtration rate (eGFR) (**c**) in patients with chronic kidney disease. Correlation studies between renal cortex SUVmean and serum creatinine (**d**), urea nitrogen (**e**), and eGFR (**f**) in patients with chronic kidney disease.

In CKD group, increased SUVmax was correlated with lower serum creatinine (*r* = −0.25, *P* = 0.015) and urea nitrogen (*r* = −0.33, *P* < 0.01). Also, increased SUVmean was correlated with lower serum creatinine (*r* = −0.23, *P* = 0.03) and urea nitrogen (*r* = −0.28, *P* < 0.01). Increased SUVmax and SUVmean were both fairly correlated with increased eGFR (*r* = 0.29, *P* < 0.01; *r* = 0.27, *P* < 0.01) (Fig. 7a–f).

### 3.9 ROC curve analyses

**Fig. 8:**
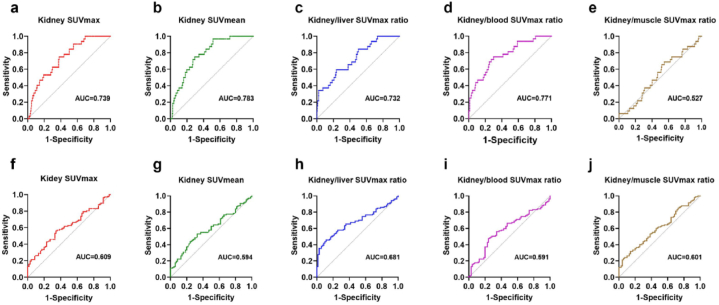
ROC curve analyses to distinguish kidney disease from normal group (**a–e**) and distinguish primary from secondary kidney disease group (**f–j**).

The ability of renal cortex SUVmax, SUVmean, kidney/liver uptake ratio, kidney/blood uptake ratio and kidney/muscle uptake ratio to distinguish kidney disease from normal group and distinguish primary from secondary kidney disease was evaluated by ROC curve analyses (Fig. 8a–j). The renal cortex SUVmax (AUC = 0.739), SUVmean (AUC = 0.783), kidney/liver uptake ratio (AUC = 0.732) and kidney/blood uptake ratio (AUC = 0.771) showed better performance in distinguishing patients with kidney disease, and the optimal cutoff values, AUCs, and P values for ROC curves were summarized in Table 5. The efficacy of using kidney/muscle uptake ratio to differentiate patients with renal disease is unsatisfactory, and using renal cortex SUVmax, SUVmean, kidney/liver uptake ratio, kidney/blood uptake ratio and kidney/muscle uptake ratio and SUVmean to distinguish between primary and secondary renal diseases is also unsatisfactory.

**Table 5:** Efficacy of metabolic parameters analyzed by ROC curve in distinguishing patients with or without kidney disease.

Variables	Cutoff value	AUC	*P* value	Sensitivity (%)	Specificity (%)
SUVmax	2.11	0.739	*P* < 0.0001	62.1	75.0
SUVmean	1.87	0.783	*P* < 0.0001	70.6	75.0
Kidney/liver uptake ratio	0.80	0.732	*P* < 0.0001	76.3	59.4
Kidney/blood uptake ratio	1.16	0.771	*P* < 0.0001	70.1	75.0

## 4 Discussion

The studies on the incidence of tumor in patients with kidney disease were predominantly concentrated on patients with late-stage kidney disease. It has been reported that the incidence of tumor in patients with late-stage kidney disease was higher than that in the general population and the incidence of tumor in patients with late-stage kidney disease was about 5 %. In our study, the overall incidence of malignant tumor in 182 patients with kidney disease was 20.9 %, and the incidence detected by PET was 18.6 %, which was significantly higher than previous studies [[9], [10], [11]]. We also found that tumor might occur in patients with various types of kidney disease, not limited to those patients with end stage renal disease (ESRD). Patients with AKD, A/C and CKD who did not progress to ESRD were all at risk, emphasizing the importance of monitoring tumor incidence in patients with various types of kidney disease. The mechanism of increased tumor risk in patients with kidney disease has not been completely clarified and the following mechanisms might explain the increased tumor incidence. Loss of renal excretion function could cause or results metabolic imbalances, aggravate oxidative stress and inflammation, and reduced renal elimination also might lead to the accumulation of carcinogenic compounds [12,13]. Pro-oxidative damage in uremia might be related with the increased tumor development introduced by the activation of chronic inflammation-related complement, cytokine production, and neutrophil aggregation [14]. In addition, peritoneal dialysis and hemodialysis might cause impaired DNA repair, which could lead to increased DNA damage of patients and further tumor formation [15].

A study in Taiwan reported that gender had an impact on the type of tumor in patients with ESRD. The three most frequent tumors in male patients with ESRD were liver, bladder and kidney cancer, while female ESRD patients most commonly suffered from bladder, kidney, and breast cancer [4]. The tumor types of ESRD patients seemed to be related to geographic regions and countries. The common tumor types among ESRD patients in Taiwan were presented above, while in the Korean population, the most commonly observed tumor sites in ESRD patients were the colorectum, lung and liver [15]. In our study, we found that among all patients with kidney disease, the three most common malignant tumors were multiple myeloma, lymphoma and lung cancer. Furthermore, the most common tumor observed in patients with AKD was also multiple myeloma. And more than half of multiple myeloma patients presented with AKD, emphasizing the importance of considering the possibility of multiple myeloma occurrence in patients with AKD. The most common cause of severe acute kidney injury in multiple myeloma patients is tubulointerstitial nephropathy, which results from very high circulating concentrations of monoclonal immunoglobulin free light chains [16].

PET/CT combines the functional and metabolic information provided by PET with the anatomical information obtained from CT, enabling comprehensive evaluation of the pathophysiological and morphological changes of the disease [17,18]. Compared to other imaging modalities such as X-ray, CT, and MRI, PET/CT imaging allows for whole-body visualization of lesions in a single scan, making it widely utilized in disease diagnosis, staging, etc. In our study, PET/CT demonstrated a tumor detection rate of 89.5 % in patients with kidney disease, exhibiting excellent diagnostic efficacy. Four malignant tumors were missed by PET/CT, including two cases of multiple myeloma and two cases of chronic lymphocytic leukemia. It has been found that false-negative PET/CT results were observed in 12 % of multiple myeloma patients, particularly in older individuals with a clinical history of smouldering multiple myeloma, and these patients exhibited lower levels of hexokinase-2 expression compared to those with positive PET/CT findings [19]. FDG uptake is usually not appreciably increased in malignant lymphocytes of some indolent lymphoid malignancies, including chronic lymphocytic leukemia/small lymphocytic lymphoma, which may be the reason for the missed diagnosis of PET/CT [20].

[^18^F]FDG is a glucose analogue and it may enrich in tissues when high glucose consumption occurs. Activated inflammatory cells also showed increased expression of glucose transporters, leading to the accumulation of [^18^F]FDG [7]. In the field of kidney, many studies have confirmed the application value of [^18^F]FDG in renal transplant patients [21,22]. There was [^18^F]FDG accumulation in renal parenchyma observed in AKI patients, which might be related to renal infiltration of inflammatory cells [23]. However, there was no study on [^18^F]FDG uptake in renal cortex of patients with and without kidney disease, as well as different types of kidney disease. The clinical value of [^18^F]FDG in the diagnosis of inflammatory lesions has been fully confirmed [24]. Our study found that there was a significant difference in [^18^F]FDG uptake in renal cortex between patients with and without kidney disease, indicating that [^18^F]FDG PET/CT can effectively identify patients with kidney disease. The incidence and mortality of AKI are high [25]. Even though the renal function of most patients can return to normal within a period of time, the risk of CKD increases in patients with unrecovered renal function, and may even progress to ESRD [26]. In our study, [^18^F]FDG in renal cortex showed potential in distinguishing AKD and CKD patients, which may help to identify the transformation of AKD to CKD and predict the prognosis of patients. According to whether there was a clear cause of kidney disease, we divided kidney disease into primary and secondary kidney disease. The [^18^F]FDG uptake of renal cortex in patients with secondary kidney disease was higher than that of primary kidney disease. But it seemed that we could not completely distinguish these two types of kidney diseases only by using the metabolic parameters of [^18^F]FDG PET/CT. The possibility that [^18^F]FDG PET/CT can determine the etiology of kidney disease needs further study.

[^18^F]FDG is mainly excreted through the kidney. In patients with impaired renal function, urinary [^18^F]FDG excretion may decrease, resulting in increased background activity [27]. However, the impact of renal function on [^18^F]FDG biodistribution is still unclear, because some studies have shown that renal function did not significantly affect the biodistribution of [^18^F]FDG, while others have reached the opposite conclusion. A prospective study involving 58 patients showed no significant difference in SUV values of the aortic blood pool, liver, spleen, lung, lymph nodes, and skeletal muscles between patients with compromised renal function and those with normal renal function [28]. Our study involving 132 patients suggested that impaired renal function did not significantly affect the clearance of [^18^F]FDG in the blood pool and liver, which was consistent with the above study. Then a study included 20 healthy volunteers and 20 patients with suspected renal failure (blood serum creatinine level >1.1 mg/dl), and found that in patients with high serum creatinine, the accumulation of [^18^F]FDG increased in blood pool, but there was no significant change in liver [27]. And a study compared the FDG uptake in liver and mediastinal blood pool of 67 patients with impaired renal function (eGFR <60 mL/min/1.73 m2) and 166 patients with normal renal function (eGFR ≥60 mL/min/1.73 m2), and found that the FDG uptake in liver and mediastinal blood pool of patients with renal insufficiency was higher [29]. The reasons for the different results may include the different research scales, different clinical features of the included cases, and the different diagnostic criteria for renal dysfunction, etc.

Fibroblast activation protein (FAP) is a membrane-anchored glycoprotein, and FAPI PET imaging has shown potential in evaluating renal fibrosis [30]. In patients with a wide range of kidney diseases, it was found that elevated FAPI uptake correlating with poorer kidney function outcomes [31,32].The correlation between renal ^68^Ga-PSMA-11 PET/CT parameters and renal function tests has been demonstrated [33]. However, the impact of renal function on [^18^F]FDG biodistribution of kidney and its clinical implications remain unclear. GFR represents the flow of plasma from the glomerulus into Bowman's space over a specified period and is a key indicator for renal function [34]. In our study, we found a positive correlation between renal cortex [^18^F]FDG uptake and eGFR in patients with CKD, while renal cortex [^18^F]FDG uptake was negatively correlated with serum creatinine and blood urea nitrogen. [^18^F]FDG enters the kidney through the renal artery, then it is filtered through the glomerulus and reabsorbed partially in the proximal tubule, finally excreted through the urine [35]. Partial proximal tubules are located in renal cortex, so impaired renal function may affect [^18^F]FDG uptake in renal cortex.

Our research has the following limitations. This is a single-center retrospective study with a relatively small sample size, which may limit the generalizability of our findings. Prospective studies with larger cohorts are needed to confirm our results. Additionally, while our study demonstrates significant group-level differences in [^18^F]FDG uptake across kidney disease classifications, individual variability within these groups poses challenges for clinical application on a patient-by-patient basis. Thus, while SUV values can distinguish disease categories at a broader level, caution is required when interpreting these values individually. Further exploration of changes in FDG biodistribution patterns in patients with renal disease could provide valuable insights for the interpretation of [^18^F]FDG PET/CT scans in this population and investigate this variability more deeply to refine diagnostic accuracy for individual cases. In addition, although [^18^F]FDG PET/CT has shown some application prospects in kidney disease, whether the benefit of radiation examination is greater than that of non-invasive and radiation-free tests such as blood tests remains to be further analyzed. Lastly, [^18^F]FDG uptake in the right psoas muscle of 8 patients with kidney disease was abnormally elevated due to inflammation and other factors, leading us to use the SUVmean of the right gluteus maximus as an alternative measurement in these cases.

## 5 Conclusion

For patients with kidney disease, [^18^F]FDG PET/CT can be used to systematically screen tumors and inflammatory lesions. To some extent, the [^18^F]FDG uptake of renal cortex may distinguish different types of kidney diseases and is correlated with renal function.

## CRediT authorship contribution statement

**Hao Jiao:** Conceptualization, Data curation, Formal analysis, Investigation, Methodology, Software, Supervision, Validation, Writing – original draft, Writing – review & editing. **Yongkang Qiu:** Writing – review & editing, Methodology, Formal analysis, Data curation. **Zhao Chen:** Writing – review & editing, Data curation. **Yongbai Zhang:** Writing – review & editing, Visualization, Data curation. **Wenpeng Huang:** Writing – review & editing, Data curation. **Qi Yang:** Writing – review & editing, Data curation. **Lei Kang:** Writing – review & editing, Funding acquisition, Data curation.

## Ethics approval and consent to participate

All procedures were performed in compliance with relevant laws and institutional guidelines and have been approved by the appropriate institutional committee. The Institutional Review Board (IRB) of Peking University First Hospital approved this retrospective study and the approval number is 2014-749. The Committee has determined that due to the retrospective nature of the study, the requirement for obtaining informed consent from the patients whose data and/or samples were used has been waived. The reasons for this decision include:

1 The study involves minimal risk to the subjects.
2 The research could not practicably be conducted without the waiver.
3 The rights and welfare of the subjects will not be adversely affected by the waiver.

Waived the need for written informed consent.

And verbal informed consent was obtained from all individual patients included in the study. All human research patients provided informed consent for publication of case details, images and any other personal information.

## Availability of data and materials

The datasets generated during and/or analyzed during the current study are available from the corresponding author on reasonable request.

## Declaration of generative AI and AI-assisted technologies in the writing process

During the preparation of this work the authors used ChatGPT to improve readability and language. After using this tool, we reviewed and edited the content as needed and take full responsibility for the content of the publication.

## Funding

This work was supported by the 10.13039/501100001809National Natural Science Foundation of China (82171970), the Beijing Science Foundation for Distinguished Young Scholars (JQ21025), the 10.13039/501100009592Beijing Municipal Science & Technology Commission (Z221100007422027), National High Level Hospital Clinical Research Funding (Interdisciplinary Research Project of 10.13039/100017415Peking University First Hospital, 2023IR17).

## Declaration of competing interest

The authors declare that they have no known competing financial interests or personal relationships that could have appeared to influence the work reported in this paper.
